# A survey of pediatricians' attitudes regarding influenza immunization in children

**DOI:** 10.1186/1471-2431-9-8

**Published:** 2009-01-30

**Authors:** Daniel J Levy, Christopher S Ambrose, Napoleon Oleka, Edward B Lewin

**Affiliations:** 1 Child & Teen Wellness Center, Owings Mills, MD, USA; 2 MedImmune, Gaithersburg, MD, USA

## Abstract

**Background:**

The Advisory Committee on Immunization Practices advocates that influenza immunization is the most effective method for prevention of illness due to influenza. Recommendations for vaccination of children against influenza have been revised several times since 2002, and as of 2008 include all children 6 months to 18 years of age. Nevertheless, influenza immunization rates have remained low.

**Methods:**

We surveyed practicing pediatricians in Maryland in the spring of 2007 to determine their attitudes and practices toward childhood influenza immunization.

**Results:**

The overall response to the survey was 21%. A total of 61% of respondents reported that immunization either is cost neutral or produces a loss, and 36.6% noted it was minimally profitable. Eighty-six percent of respondents were receptive to supporting school-based immunization programs, and 61% indicated that they would participate in such programs. Respondents reported higher rates of immunization of select patient groups than those noted by the Centers for Disease Control and Prevention

**Conclusion:**

Vaccination was reported to occur at multiple types of patient encounters, as recommended. Survey respondents stated that practice-based immunization was not a profitable service. Pediatricians were supportive of school-based immunization programs, and more than half stated they would be actively involved in such programs. School-based programs may be critical to achieving high vaccination coverage in the school-aged population.

## Background

Influenza causes annual epidemics and affects all segments of the population. Children experience the highest rates of infection, shed the greatest quantities of influenza virus for extended periods of time, and have long been recognized as vectors for spread of disease [1-4]. Young children are also at increased risk of complications from influenza. Because of high rates of influenza-related hospitalizations in children younger than 24 months of age, the Advisory Committee on Immunization Practices (ACIP) encouraged universal vaccination of children aged 6 to 23 months in 2002 [5]. In 2004, the ACIP made a formal recommendation for universal vaccination among children 6 to 23 months [6]. Later, this recommendation was expanded to children 6 to ≤ 59 months of age [7]. Recommendations by the ACIP for children were subsequently further expanded and as of 2008 included the following groups: all children 6 months to 18 years of age, children with certain medical conditions, children who are contacts of persons at higher risk for complications due to influenza [4]. In addition, ACIP recommends vaccination for all persons, including school-aged children, who want to reduce the risk of becoming ill with influenza or of transmitting influenza to others [4].

Despite these recommendations, estimates of influenza vaccination levels reported by the US Centers for Disease Control and Prevention (CDC) fall below targets proposed in the Healthy People 2010 initiative [4,8,9]. Possible reasons for low rates of influenza vaccination may be limited practitioner recognition of the severity of influenza in young children, difficulty in identifying appropriate high-risk candidates, confusion about which provider is responsible for immunization when multiple providers are involved in patient care, and underutilization of strategies known to improve vaccination rates [10,11].

This study was designed to determine the attitudes and practices of pediatricians regarding immunizing children against influenza.

## Methods

The Maryland Chapter of the American Academy of Pediatrics consists of 1100 members, of which 900 maintain a current practice. The 900 practicing pediatricians in the state were issued a survey by mail during the spring of 2007 to determine their attitudes and practices regarding childhood influenza vaccination, based on their own opinions and personal recollections. Thirteen questions were selected for inclusion in the questionnaire [see additional file 1] and were divided into 4 major categories: size and location of practice (questions 1, 2, 5); influenza immunization practices regarding patient selection and specific type of vaccine administered (questions 3, 3a, 4, 12, 13); profitability of immunization (questions 6, 7); and participation in school-based influenza immunization programs (questions 8–11).

Questions were free response, multiple choice, or simple yes or no; more than 1 answer could be selected for some multiple-choice questions. Data were tabulated based on the number of responses for each choice per individual question divided by the total number of responses for that question. Free text responses to question 7 were subjectively categorized and the percentage of responses in each category was calculated. Clinicians were provided with a return envelope, were sent weekly e-mail reminders to prompt them to return the survey by a specified cutoff date, and were compensated $10 for completing the survey. The survey was coordinated by the Maryland Chapter of the American Academy of Pediatrics, and all results were tabulated and analyzed by the sponsor (MedImmune, Gaithersburg, MD).

## Results

### Response rate

Of the 900 pediatricians who were surveyed, a total of 190 questionnaires were returned and analyzed, for an overall response rate of 21.1%. Some of those who replied did not provide responses to all questions. Responses were balanced by sex and practitioners spanned a 66-year range in age, with the median age being younger than 50 years (Table 1).

**Table 1:** Responses by Pediatricians Surveyed in Maryland to a Questionnaire Regarding Attitudes Regarding Influenza Immunization Practices

**Demographics of respondents**	
Median age of practitioners, y (range)	48 (25–91)
Sex, %	
Men	50.3
Women	49.7
**Size and location of practice**	
Location, %	
Suburban	54.1
Urban	37.2
Rural	6.0
Combination	2.7
Practice size, n	
Median (range)	6000 (30–80,000)
Mean	8797
VFC-eligible children in practices, %	
Median (range)	20 (0–100)
Mean	32
**Influenza immunization practices**	
Patients in at-risk categories immunized with influenza vaccine, %	
Children aged 6–23 mo	75
Children aged 24–59 mo	50
Children at high risk	80
Household contacts of at-risk individuals	45
When and where immunization occurs, %	
Regular visits	98.8
Sick visits	74.6
Special infkuenza immunization clinics	72.2
Availability of callback system, %	39.8
Vaccine types used, %	
Thimerosal-free inactivated, median (range)	50 (0–100)
Thimerosal-containing inactivated, median (range)	50 (0–100)
Live attenuated nasal spray, median (range)	5 (0–100)
How much more burdensome would it be to ask if the parent or a provider ever noted wheezing or asthma in the child (5-point scale; 1 = not at all and 5 = very), %	
1	54.2
2	26.0
3	11.3
4	5.1
5	3.4
**Profitability of influenza immunization**	
How profitable is influenza immunization of children?, %	
Cost neutral	46.5
Produces a loss	14.5
Minimally profitable	36.6
What would improve profitability?, %	
Better reimbursement	56.5
Better payment for vaccine administration	13.7
Less costly vaccine	8.7
All other responses	21.1
**Participation in school-based immunization programs**	
Would you support a school-based immunization program?, %	85.7
Would you participate in a school immunization program?, %	60.8
What would persuade you to participate?, %	
Financial remuneration	57.5
Civic involvement	53.1
Source of new patients	22.9
Nothing	22.3
How might you participate?, %	
Off-site consultation	69.5
On-site supervision	52.5
Both on-site and off-site	38.4

### Size and location of practice

A little more than one third of practices were located in urban areas, approximately one half were situated in suburbs, 6% were based in rural areas, and <3% were in a combination of areas. The median practice was 6000 patients, of which fewer than one third, on average, was eligible for the Vaccines for Children (VFC) program (Table 1).

### Influenza immunization practices

For all age groups and specified at-risk candidates, the percentage of patients reportedly immunized by respondents exceeded national averages reported by the CDC [4]. With respect to the setting for immunization, nearly all practitioners reported immunizing patients during regular visits, and approximately three quarters also reported vaccinating during sick visits and at special influenza vaccine clinics. Fewer than half of all practices had any form of callback system to contact at-risk candidates who had not yet been immunized (Table 1).

Various influenza vaccines are marketed, including inactivated preservative-free formulations in single-dose prefilled syringes; inactivated thimerosal-containing formulations in multidose vials; and a live attenuated, preservative-free, single-dose nasal spray. Concerning the specific types of vaccine administered, those who responded noted that inactivated influenza vaccines were administered more frequently than the nasal spray. Use of inactivated vaccines was evenly split between the thimerosal-free and thimerosal-containing formulations. Providers with more VFC-eligible children were more likely to administer thimerosal-free vaccine, and those with fewer VFC-eligible children were more likely to administer thimerosal-containing inactivated vaccine (Table 1).

Pediatricians were queried to determine how burdensome it would be to ask, in addition to other standard vaccination screening questions, whether the parent or healthcare provider had ever noted asthma or wheezing in individual children, conditions which are potential warnings/precautions for the administration of the live attenuated nasal spray influenza vaccine. Based on a 5-point scale with 1 being "not at all" and 5 being "very," approximately 80% of those who replied noted that it would not be a burden at all, or only a very slight burden, whereas 3.4% specified that it would be very burdensome to ask this additional question (Table 1).

### Profitability of immunization

Overall, pediatricians reported that influenza immunization is not a profitable service. A total of 61% of respondents reported that it either is cost neutral or produces a loss; 36.6% noted it was minimally profitable. As noted by two thirds of responses, the most significant barrier to profits is poor reimbursement for costs of the vaccine and administration. Acquisition price of the vaccines was not seen as a major obstacle (Table 1).

### Participation in school-based immunization programs

Eighty-six percent of respondents were receptive to supporting school-based immunization programs, 61% indicated that they would participate in such programs. Of those who would participate, about 70% noted that they would provide off-site consultation, half were receptive to being available on-site in a supervisory role, and approximately one third would be willing to provide both on-site and off-site services. Primary incentives for participation were financial remuneration and civic involvement. Providers with high VFC-eligible populations were more likely to state that they would participate in school-based programs (76% of providers with ≥ 50% VFC-eligible populations would participate compared with 53% of those <50% VFC-eligible); however, providers with high and low VFC-eligible populations expressed similar overall support of such programs (Table 1).

## Discussion

Various provider groups have been surveyed regarding their knowledge of recommendations for influenza immunization [10-12]. Not surprisingly, pediatricians tend to be the most knowledgeable with respect to current recommendations for children, and this is the group that was targeted in the present study.

Current recommendations state that influenza vaccination should be offered during routine healthcare visits, sick visits, and influenza vaccine clinics, among other venues [4]. The survey results support that regular visits, sick visits, and special clinics are regularly used by pediatricians. Pediatricians noted that they administer an injectable form of influenza vaccine more frequently than the nasal spray. In Maryland, the 2 largest medical insurers did not reimburse for the nasal spray at the time of the survey and this likely influenced vaccine choice. Despite controversy over the safety of thimerosal-containing vaccines, preservative-containing and preservative-free injectable vaccines were equally used; VFC participation appeared to increase utilization of thimerosal-free formulations. Similar to previous reports [11], the majority of practices did not have any callback system in place to notify patients about immunization opportunities. Respondents reported that they immunize children at rates in excess of those reported by the CDC from national surveys [13], likely due to overestimation of their actual vaccination rates [11].

Overall, pediatricians do not believe the practice of administering influenza vaccine to children is profitable for their practice. Increased reimbursement for influenza vaccine and its administration would likely increase vaccination coverage in the future.

Almost 90% of respondents noted they would support school-based immunization programs, the value of which has been previously demonstrated [14-17]; approximately 60% stated they would participate in such programs. Given the logistical obstacles to vaccinating large numbers of school-aged children, school-based vaccination programs may be essential for achieving high rates of vaccination coverage in children 5 to 18 years of age, who are recommended to be vaccinated beginning in the 2008–2009 influenza season [4].

There are several inherent limitations to the survey findings. Results of previous surveys of immunization practices among various physician groups indicate that pediatricians are fairly diligent in providing feedback, and response rates of 50% to 60% are common [10,11,18]. It is unclear why the response rate was less than 25% in the present study. There is potential for bias given this response rate; responders may have disproportionate interest in influenza vaccination. Because only pediatricians in the state of Maryland were surveyed, extrapolations to broader populations are problematic. In particular, findings regarding school-based programs may have been influenced by past school-based influenza vaccination programs conducted in Maryland [14,15,17]. Nevertheless, several of the findings pertaining to immunization practices are consistent with survey results from other investigators [11].

## Conclusion

Vaccination was reported to occur at multiple types of patient encounters, as recommended. Survey respondents stated that practice-based immunization was not a profitable service. Pediatricians were supportive of school-based immunization programs, and more than half stated they would be actively involved in such programs. School-based programs may be critical to achieving high vaccination coverage in the school-aged population.

## Competing interests

Dr. Levy has served on an advisory panel for MedImmune; Drs. Ambrose, Oleka, and Lewin were employees of MedImmune at the time of the study. Drs. Ambrose and Oleka are current employees of MedImmune.

## Authors' contributions

DJL helped design the survey, conducted the study, and aided in the analysis of the study results. CSA aided in the analysis of the study results and helped draft the manuscript. NA helped design the survey and tabulated and analyzed the survey results. EBL designed the survey and aided in the analysis of study results. All authors read and approved the final submitted manuscript.

## Pre-publication history

The pre-publication history for this paper can be accessed here:



## Supplementary Material

Additional file 1 **Levy Supplementary File.** Pediatrician Survey – The survey distributed to practicing pediatricians in Maryland is presented. Click here for file
