# Supplementary material for: A survey of pediatricians' attitudes regarding influenza immunization in children
Source: BMC Pediatr. 2009 Jan 30;9:8. doi: 10.1186/1471-2431-9-8 (PMC2645391; doi:10.1186/1471-2431-9-8)
Supplement: Additional file 1 — Levy Supplementary File. Pediatrician Survey – The survey distributed to practicing pediatricians in Maryland is presented. [file 1471-2431-9-8-S1.pdf]

AMERICAN ACADEMY OF PEDIATRICS—MARYLAND CHAPTER

Questionnaire to Determine Attitudes and Practices Regarding Immunizing Children Against Influenza

1. Is your practice?: ____ urban ____suburban ____rural

2. Approximately how many patients does your practice serve? _______

3. Estimate the percent of patients in the following at-risk categories you immunize with flu vaccine who are:

   a. Children aged 6-23 months ____

   b. Children aged 24-59 months ____

   c. Children at high-risk ____

   d. Children who are household contacts of high-risk individuals ____

3a. Do you have a callback system for flu immunization? Yes ____ No ____

4. Do you immunize patients during:

   a. Regular visits Yes____ No____

   b. Special influenza immunization clinics Yes____ No____

   c. Sick visits Yes____ No____

   [Respondents can select more than one category]

5. What percent of children in your practice are VFC eligible? _____

6. Is influenza immunization profitable for your practice?

   a. Significantly profitable ____

   b. Minimally profitable ____

   c. Cost neutral ____

   d. Produces a loss ____

7. What would improve profitability? ____________________________________

8. Would you be supportive of a school-based immunization program? Yes___ No___

9. Would you participate in a school immunization program? Yes___ No___

10. What, if anything, would persuade you to participate?

a. Financial remuneration ____

b. Civic involvement ____

c. Source of new patients ____

d. Nothing ____

11. How might you participate?

a. On-site supervision ____

b. Off-site supervision ____

12. What percentage of the following vaccines do you use in your practice?

a. Thimerosal-free injectable inactivated _____

b. Thimerosal-containing multiple dose injectable inactivated _____

c. Nasal spray live attenuated _____

13. FluMist is presently being considered by the FDA for supplemental licensure for children 1-5 years of age without a history of wheezing or asthma. You are already asking a number of questions before giving any influenza vaccine to children (e.g., history of egg allergy or Guillan-Barre Syndrome). How much more burdensome would it be to ask the following additional question? (1 = not at all; 5 = very)

Has the parent or healthcare provider ever noted wheezing or asthma in this child?

1____ 2____ 3____ 4____ 5____
